# Autistic children at risk of being underestimated: school-based pilot study of a strength-informed assessment

**DOI:** 10.1186/s13229-015-0006-3

**Published:** 2015-03-06

**Authors:** Valérie Courchesne, Andrée-Anne S Meilleur, Marie-Pier Poulin-Lord, Michelle Dawson, Isabelle Soulières

**Affiliations:** Rivière-des-prairies Hospital, Centre d’Excellence en Troubles Envahissants du Développement de l’Université de Montréal (CETEDUM), 7070 boulevard Perras, Montréal, QC H1E 1A4 Canada; Psychology Department, Université du Québec à Montréal, C.P. 8888 succursale Centre-ville, Montréal, H3C 3P8 Canada

**Keywords:** Autism, Assessment, Intelligence, Perception, Cognition

## Abstract

**Background:**

An important minority of school-aged autistic children, often characterized as ‘nonverbal’ or ‘minimally verbal,’ displays little or no spoken language. These children are at risk of being judged ‘low-functioning’ or ‘untestable’ via conventional cognitive testing practices. One neglected avenue for assessing autistic children so situated is to engage current knowledge of autistic cognitive strengths. Our aim was thus to pilot a strength-informed assessment of autistic children whose poor performance on conventional instruments suggests their cognitive potential is very limited.

**Methods:**

Thirty autistic children (6 to 12 years) with little or no spoken language, attending specialized schools for autistic children with the highest levels of impairment, were assessed using Wechsler Intelligence Scale for Children (WISC-IV), Raven’s Colored Progressive Matrices board form (RCPM), Children’s Embedded Figures Test (CEFT), and a visual search task. An age-matched control group of 27 typical children was also assessed.

**Results:**

None of the autistic children could complete WISC-IV; only six completed any subtest. In contrast, 26 autistic children could complete RCPM, with 17 scoring between the 5th and 90th percentile. Twenty-seven autistic children completed the visual search task, while 26 completed CEFT, on which autistic children were faster than RCPM-matched typical children. Autistic performance on RCPM, CEFT, and visual search were correlated.

**Conclusion:**

These results indicate that ‘minimally verbal’ or ‘nonverbal’ school-aged autistic children may be at risk of being underestimated: they may be wrongly regarded as having little cognitive potential. Our findings support the usefulness of strength-informed approaches to autism and have important implications for the assessment and education of autistic children.

## Background

Autistic children^a^ who reach school age with little or no spoken language, and thus acquire labels such as ‘nonverbal’ or ‘minimally verbal’, have recently attracted concern as a neglected subgroup in autism research [1]. Many such children are judged ‘low-functioning’ or ‘untestable’ through conventional assessments of cognitive abilities, on which they may not achieve even a basal score. Therefore, their potential is estimated to be extremely limited. At a time when very early development dominates autism research priorities [2,3] and is widely claimed to be determinative, the difficulties faced by autistic children so situated raise important concerns. Not only are they likely to be regarded and treated as though very low-functioning, they are in addition considered far too old for popular interventions to improve their outcomes [4]. The possibility that the cognitive potential of some, many, or most of these autistic children is at risk of being underestimated thus merits attention.

Current expert opinion recommendations for assessing minimally verbal school-aged autistic children emphasize ‘core domains’ (language, social behaviors, repetitive behaviors), typicality (in development and range of abilities), and comprehensiveness [5]. However, this kind of assessment may not be practical due to limited resources, and further, may not alleviate the risk of being underestimated faced by children whose developmental paths and range of abilities are highly atypical. Indeed, many minimally verbal autistic children are characterized by marked atypicality and existing findings suggest they may be disadvantaged by tests which require typicality, such as commonly used Wechsler-type intelligence tests and Vineland-type adaptive or developmental tests [6,7]. Even tests considered ‘well-suited for use with minimally verbal children’ ([5]; Tables one to six), such as picture vocabulary tests, may require specific typical abilities, such as the ability to reliably point, which some or many nonspeaking autistic children may lack [8].

An overlooked approach, in the alternative, is to engage current knowledge of autistics’ atypical cognitive strengths ([9], for a review) when assessing the potential of older autistic children who speak very little or not at all. This resembles approaches to other disabilities (for example, blindness) where skills and abilities may present in highly atypical spontaneous and learned forms (for example, echolocation, braille reading, rapid speech recognition; [10-12]), and where adherence to conventional assessments of cognitive ability would have major detrimental consequences. Our aim was therefore to pilot a strength-informed assessment for minimally verbal school-aged autistic children who are ‘untestable’ or perform poorly in conventional assessments.

Because this is a novel approach, and respecting the problem of limited resources in many school-based settings, we chose as priority a small number of easily administered tests for this pilot study. The first test is Raven’s Progressive Matrices, an important test of general and fluid intelligence [13] on which autistics have displayed an advantage over Wechsler scales of intelligence that is not found in the nonautistic population [6,7,14-16]. All versions of Raven’s Matrices are relatively rapid and simple to administer, a priority in contexts with limited resources. We chose to use the board form of Raven’s Colored Progressive Matrices (RCPM; [17]), given the age of the population to be tested (6 to 12 years) and our objective to assess autistic children who are conventionally difficult to test or untestable. While all Raven versions minimize both the need for instructions and demands for specific abilities (for example, typical language comprehension or production), RCPM board form enhances this Raven feature and, further, eliminates any requirement to point. There is preliminary evidence [18] that an improvised board or ‘puzzle’ form of RCPM, while equivalent to the on-paper version in typical children, produces both better scores and a higher completion rate in school-aged autistic children labeled with severe intellectual disability. A published large data set including RCPM board form scores for 256 autistic children aged 7 to 11 years [19] also suggests this test belongs in a strength-informed assessment for minimally verbal autistic children in this age range.

We chose visual search [20,21] and embedded figures [22-24] tasks as the second and third tests in the pilot assessment. For both kinds of tasks, which are relatively simple to administer, there are numerous reported replications of superior autistic performance [25]. As with RCPM board form, both visual search and embedded figures tasks minimize or eliminate the need for instructions, for typical language comprehension and production, and for pointing. Both tasks are considered perceptual, which respects the documented association between perception and intelligence especially prominent in autistics, but also found in the nonautistic population [15,16,26,27].

Accordingly, the aim of the present study was to reassess the cognitive potential of minimally verbal school-aged autistic children who present with a high level of impairment and are conventionally labeled as ‘low-functioning’. To do so, we piloted a simple three-part assessment better suited to autistic cognition. The results of this proposed strength-informed assessment were compared to conventional testing on Wechsler scales of intelligence and, where available, to previously recorded test results for the reassessed autistic children. Their results were also compared to an age-matched group of typical children who were similarly tested.

## Methods

### Participants

Autistic participants were recruited in two Montreal-area specialized public schools for autism spectrum disorders and exceptional needs. Children in both schools had all failed to be integrated in regular or other specialized schools; they were regarded as ‘low-functioning’ and as having important deficits in adaptive behaviors. All families of children aged from 6 to 12 years and with an autism spectrum diagnosis (39 children) in both schools were approached for their child’s participation in this study. Written informed consent to participate was given for 30 autistic children (77%). A review of the 30 participants’ files indicated an autism diagnosis based on Autism Diagnostic Observation Schedule (ADOS; [28]) for six participants; combined ADOS and Autism Diagnostic Interview Revised (ADI-R; [29]) for 12 participants; Childhood Autism Rating Scale (CARS; [30]) for two participants; and DSM-IV and expert clinician opinion for ten participants.

Autistic participants’ spoken expressive language levels, as documented by an interview with the participant’s speech therapist, were distributed as follows: 12 children with no meaningful words, ten children with isolated meaningful words, and eight children with fewer than five two-word phrases (as defined in the ADI-R: must include a verb, for example, ‘want juice’, and must not be considered echolalia). It was also noted throughout the study that many of the autistic participants could not point.

A comparison group of 27 typically developing nonautistic children matched on age and gender was recruited in an elementary school serving children from comparable socioeconomic status. Comparison participants and their first-degree relatives were screened through a semi-structured interview conducted with the parents for history of developmental, neurological, or psychiatric conditions. This procedure led to the exclusion of three children from a total sample of 30 children (one presenting with epilepsy, one having a language disorder, and one having a possible attention deficit disorder). Sociodemographic characteristics of the participants are presented in Table 1. This study was approved by the ethics committee of Riviere-des-Prairies Hospital in Montreal and the school boards of the three participating schools.

**Table 1:** **Sociodemographic characteristics of participants**

	**Autistics**	**Controls**	***P***
Number	30	27	-
Age in years	*M* = 9.36, range 6 to 12	*M* = 9.08, range 6 to 12	0.54
Gender	*M* = 21, *F* = 9	*M* = 20, *F* = 7	-
FSIQ	Non-evaluable	*M* = 96.69 (SD = 12.99)	*-*

FSIQ, Full Scale Intelligence Quotient.

### General procedure

Autistic children were individually tested in a familiar room located in their school, by one of four examiners with two or more years of clinical experience working with autistic children. Eight autistic children were evaluated in their classroom in order to diminish any anxiety caused by routine modification. In order to obtain the child’s optimal performance, session duration was adjusted to each child after consulting his teacher. The number of sessions required for completing the testing varied from one to six (*M* = 3.8; SD = 1.45), and their duration ranged from 15 to 25 minutes. When necessary, an educator familiar to the child was present. Also when necessary, to avoid disruptions to entrenched routines, autistic children were reinforced as usual by their educator (for correct responses in practice trials; for any response in test trials). However, it should be noted that while autistic children were assumed to be dependent on reinforcers in their education programs, they often ignored or refused offered reinforcers while being tested.

Nonautistic children were similarly tested in their school, by the same examiners and with the length of each evaluation session (usually two per child) adapted to the attention capacities of each child. For all children, the four tests were administered in a counterbalanced order.

### Conventional assessment

#### Wechsler Intelligence Scale for Children, Fourth Edition

In common with all Wechsler scales of intelligence, WISC-IV [31] estimates general intelligence and its components (in WISC-IV, these are verbal comprehension, perceptual reasoning, processing speed, working memory) through a battery of subtests assessing specific skills which are thought to reflect latent abilities. All WISC-IV subtests that do not require a verbal response (block design, matrix reasoning, picture concepts, coding, and symbol search) were attempted with the autistic children. All subtests included in WISC-IV Full Scale IQ (block design, similarities, digit span, matrix reasoning, coding, vocabulary, letter-number sequencing, picture concepts, symbol search, and comprehension) were administered to the nonautistic children.

#### Leiter-Revised

Leiter-R is a nonverbal intelligence test designed to assess children with language difficulties. Seven of the autistic children had previously completed the Leiter-R Visualization and Reasoning Battery [32], which had been administered by a school psychologist; these scores were collected as available.

### Strength-informed assessment

#### Raven’s Colored Progressive Matrices board form

RCPM board form [33] is a one-format 36-item test divided into three sets of 12 items (A, Ab, B) which increase in difficulty and complexity within and across sets. Each item is composed of a pattern or a two-by-two matrix with the last piece missing, leaving an empty hole or space in the board. There are six movable pieces underneath, among which the one that best completes the matrix must be chosen to fill the empty space. Sets A and B of RCPM are the same as sets A and B from Raven’s Standard Progressive Matrices [17].

The first problem was presented to the child without oral instructions. The examiner simply pointed to the empty space in the matrix. If the child did not understand the task (for example, stacking the pieces, or trying the entire series of pieces without choosing one), he was trained until he understood the task, which was made evident by the production of a correct or incorrect placement of a single piece in the empty space.

This training began by presenting a completed 12-piece wooden jigsaw puzzle to the child. The examiner then removed a piece of the puzzle in front of the child and prompted him to put it back in the corresponding empty space with a gesture alternately showing the empty space and the corresponding removed piece. All children who required training understood this first step. The examiner then removed a piece of the puzzle and placed it with two incorrect pieces belonging to a different puzzle. Then, if necessary, the child was once again prompted to select the one correct piece among these three pieces, and place it in its corresponding empty space. Several trials with three different alternative puzzles were completed, until the child consistently chose the correct piece. Lastly, practice trials were conducted using six homemade matrices similar to the easiest RCPM items.

#### Visual search

**Figure 1:**
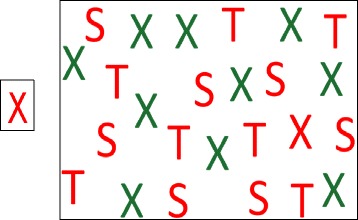
**Example of visual search test trial.** Conjunctive search with 25 distracters; the target figure given to the child is on the left.

In visual search tasks, a predetermined target must be found within a field of distracters. The test used here was an adapted cardboard form of the computerized version in O’Riordan, Plaisted, Driver, and Baron-Cohen [34]. Three different letters were successively used as targets in three set sizes (5, 15, or 25 distracters). In the feature condition, the target letter had nothing in common with the distracters (different color and shape). In the conjunction condition, the target shared one feature with each of the distracters (either shape or color). There were six trials for each set size (5, 15, or 25 distracters) and condition (feature or conjunction) for a total of 36 trials presented in random order. The target and distracters were created in Calibri font size 115 (approximately 1.8 × 2.7 cm). Targets were printed on a 3 × 2.4 cm cardboard and distracters were presented on a 28 × 21.5 cm plasticized sheet (see Figure 1).

Testing began by giving the cardboard target to the child. Then a sheet showing the target among the distracters was placed in front of him. Four easy practice trials with only one or two distracters initiated the testing. Time taken to place the cardboard letter on the corresponding target letter was manually recorded.

#### Children’s Embedded Figure Test (CEFT)

**Figure 2:**
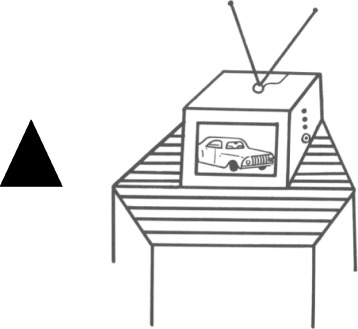
**Example of a trial in the CEFT.** The target figure given to the child is on the left.

The Children’s Embedded Figure Test (CEFT; [35]) consists of finding a target figure ‘hidden’ by its embedding in a larger meaningful pattern. There are 14 practice trials and 25 test trials (see Figure 2). The target was first given to the child and then the display was placed in front of him. A gesture toward the correct answer was used as a prompt in the practice trials when necessary. The number of targets found and the time to the correct placement of the cardboard figure on the target were manually recorded. The instruction not to turn the target figure, which is normally given to the child prior to administration, was removed for all participants. This decision was made because the autistic children could not understand this instruction. Indeed, many of the autistic children strategically turned the target in order to find it hiding within the larger pattern, showing spontaneous understanding of the disembedding task demand.

## Results

### Conventional testing

#### WISC-IV

Only six autistic children could complete any WISC-IV subtest. One autistic child completed four subtests (block design, picture concepts, matrix reasoning, coding), with scaled scores ranging from 1 to 10, and a Perceptual Reasoning Index standard score of 66. Three autistic children completed two subtests (block design and matrix reasoning) with scaled scores ranging from 1 to 15—this high score on block design; and two autistic children completed only one subtest, matrix reasoning, with scaled scores of 11 and 12. Thus, the WISC-IV subtest completed by the highest number of autistic children was matrix reasoning, but it was completed by only six children or 20% of the sample. Only one autistic child, or 3.3% of the sample, was able to achieve a WISC-IV index score, and this score was below 70. For details of WISC-IV scores for the autistic children, see Table 2.

**Table 2:** **Age and scores of nine autistic participants able to complete any WISC-IV subtest, or Leiter-R**

**Participant**	**Age**	**Block design**	**Matrix reasoning**	**Picture concepts**	**Coding**	**PRI**	**Leiter-R (standard; pc)**	**RCPM (raw; pc)**
1	10:3	10	3	1	3	66	79; 8	29; 63
2	9:2	8	7	-	-	-	93; 32	31; 83
3	7:2	15	10	-	-	-	-	27; 90
4	10:4	5	1	-	-	-	61; 0.5	22; 19
5	7:6	-	11	-	-	-	84; 14	29; 90
6	9:3	-	12	-	-	-	-	32; 83
7	9:5	-	-	-	-	-	69; 2	8; <5
8	8:8	-	-	-	-	-	56; 0.2	13; 7
9	11:2	-	-	-	-	-	45; 0.01	16; <5

*Note:* Age is years:months. Standardized WISC-IV subtest (*M* = 10; SD = 3) and PRI (*M* = 100; SD = 15) scores were obtained using Canadian norms. Leiter-R (Visualization and Reasoning Battery) scores (*M* = 100; SD = 15; percentiles) were obtained using American norms. PRI, Perceptual Reasoning Index; RCPM, Raven’s Colored Progressive Matrices; pc, percentile.

All 27 nonautistic comparison children completed all WISC-IV subtests; as recorded in Table 1, their mean full-scale IQ was 96.69 (SD = 12.99).

#### Leiter-R

Leiter-R scores for the Visualization and Reasoning Battery were available for seven autistic participants, or 23% of the sample. The test had been administered by one of the school psychologists within the year prior to the present study. Standard scores ranged from 45 to 93; the three children with standard scores higher than 70 achieved higher RCPM than Leiter-R percentile scores. For details, see Table 2, and for further comparison with and relation to RCPM scores, see below.

### Strength-informed assessment

Twenty-seven of the 30 autistic children could complete at least two of the three tests in the strength-informed assessment, and 25 of 30 could perform all three tests. Three autistic children (two boys and one girl, aged 6:0, 7:1, and 11:3, respectively) could not be tested due to apparent anxiety or other indications of distress or difficulty.

#### RCPM board form

**Figure 3:**
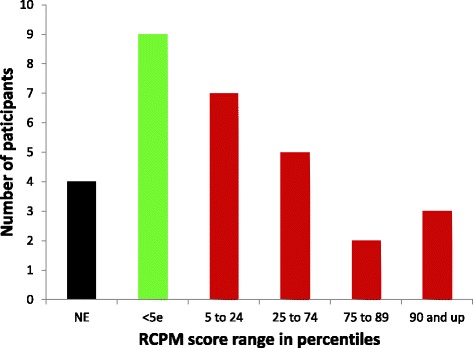
**RCPM percentile score range distribution for the 30 autistic participants.** *NE* = non-evaluable.

We obtained scores for 26 of 30 autistic participants, or 87% of the sample. Scores ranged from the 2nd percentile (estimated; see [17]) to the 90th percentile, with an *N* = 26 group percentile of 13, which corresponds to a mean IQ of approximately 83. Seventeen (65%) of the 26 children with RCPM scores performed in the normal range, that is, at or above the 5th percentile, or an estimated IQ of 75 or higher. Eight autistic children (31% of those with scores) performed at or above the 50th percentile, and three were at the 90th percentile (see Figure 3). For the 26 tested autistic children, mean raw score (out of 36) was 18.61 (SD = 8.00, range 8 to 32).

Using ANOVA, we found that the autistic children’s RCPM performance differed according to their reported spoken language level (*F* (2, 23) = 6.96, *P* < 0.005). *Post hoc* comparisons using Tukey showed that autistic children using two-word phrases performed better on RCPM (raw score *M* = 25/36; SD = 8.49) than those using no words at all (*M* = 12.75/36; SD = 3.73). Those using isolated words (*M =* 18.2/36; SD = 6.63) did not differ significantly from the two other groups (*P* > 0.10). There was no age difference between the language level groups (*P* = 0.18). Nonparametric analyses using percentiles were also carried out and led to similar results.

For the seven autistic children with Leiter-R Visualization and Reasoning Battery percentile scores (see Table 2), these were significantly lower (*Md* = 2) than their RCPM percentile scores (*Md* = 19)(*z* = −2.2, *P* < 0.05), with a large effect size (*r* = 0.59). However, performance on the two tests was strongly correlated (Spearman’s rank correlation; *r* = 0.82, *P* < 0.05).

The nonautistic comparison children obtained RCPM scores ranging from the 22nd to the 98th percentile, with a group percentile of 63, which corresponds to a mean IQ of approximately 105. Their mean raw score was 28.5/36 (SD = 4.4; range 21 to 36).

#### Visual search

Twenty-seven of the 30 autistic children completed the visual search task without requiring any prompting or explanation. Most autistic participants completed the task without seeking or accepting their usual reinforcement and without showing any behavior indicating fatigue or boredom (getting up to leave the room, putting their head on the table, pushing the material away, and so on). Interestingly, it was observed by their educators that some autistic children were able to concentrate on this task longer than on any other activity in their usual school schedule.

Both autistic and nonautistic groups performed at ceiling in number of targets found. A Group × Condition (feature vs conjunction) × Set size (5, 15, or 25) ANOVA on response time revealed a main effect of group, *F* (1, 51) = 15, 59, *P* < 0.0005, with autistic children showing slower response time (*M* = 2.56 seconds; SD = 1.3) than nonautistic children (*M* = 1.49 seconds; SD = 0.52). There was also a main effect of condition, *F* (1, 51) = 42.77, *P* < 0.0005, and a main effect of set size *F* (2, 50) = 42.30, *P* < 0.0005. The only significant interaction was between condition and set size, indicating that the magnitude of the difference between conditions increases with the number of distracters.

**Figure 4:**
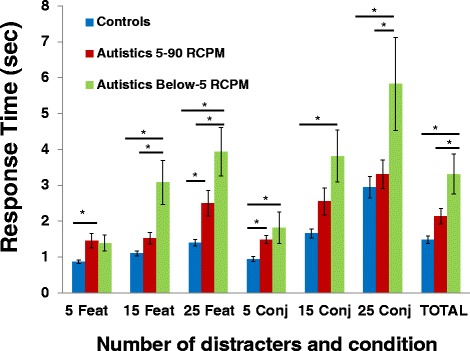
**Mean visual search response times.** Results shown are for the below-5 RCPM autistic subgroup (*N* = 9), the 5-90 RCPM autistic subgroup (*N* = 17), and typical control group (*N* = 27), for each condition (5, 15, and 25 distracters; feature and conjunctive) and the total for all trials. Asterisk represents *P* < 0.01.

The autistic group was then separated in subgroups: those who scored between the 5th and 90th percentile on RCPM (the ‘5-90 RCPM’ subgroup, *N* = 17), and those who scored below the 5th percentile on RCPM (the ‘below-5 RCPM’ subgroup, *N* = 9). Both autistic subgroups were then compared to the nonautistic group. An ANOVA revealed that the groups significantly differed from one another on the visual search time (*F* (2, 49) = 13.17, *P* < 0.001). The effect size was large: *η*_p_^2^ = 0.35. *Post hoc* comparisons using the Tukey honest significant difference (HSD) indicated that the 5-90 RCPM autistic subgroup (*M* = 2.14 seconds; SD = 0.90) and nonautistic children (*M* = 1.49 seconds; SD = 0.52) were significantly faster than the below-5 RCPM autistic subgroup (*M* = 3.31 seconds; SD = 1.67). More importantly, visual search performance of the 5-90 RCPM autistic subgroup did not differ significantly from that of the nonautistic children (*P* = 0.07) (see Figure 4).

Different subgroups matched on RCPM scores were then created by removing data from autistic children with the lowest RCPM scores and from nonautistic children with the highest RCPM scores until RCPM mean raw scores were equivalent (autistics: *M* = 24.8/36; SD = 6.4; controls: *M* = 27.3/36; SD = 3.8; *P* = 0.23). This procedure led to RCPM-matched subgroups of 13 autistic and 13 nonautistic children also matched on age (autistics: *M* = 8.92 years; SD = 1.64; controls: *M* = 9.51 years; SD = 1.12; *P* = 0.29). When comparing these RCPM-matched subgroups, it was found that autistics did not differ from nonautistics on visual search response time (*P* = 0.58).

Furthermore, there was a strong negative correlation between visual search response time and RCPM performance for the autistic children (*r* = −0.67, *P* < 0.001) indicating that the faster the participant was on the visual search task, the better he was on RCPM. This correlation was nonsignificant in the control group (*r* = −0.25, *P* = 0.23). Correlations were done while controlling for age, because there are no age-stratified norms for the visual search task.

#### CEFT

**Figure 5:**
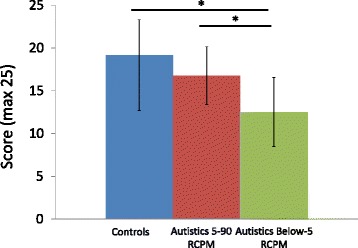
**CEFT mean score.** Number of correct responses for the below-5 RCPM autistic subgroup (*N* = 9), the 5-90 RCPM autistic subgroup (*N* = 17), and controls (*N* = 27). Asterisk represents *P* < 0.01.

Twenty-six of 30 autistic children were able to perform the CEFT. At the group level, autistic children found fewer hidden figures (*M* = 15.35; SD = 3.99) than nonautistic children (*M* = 18.19; SD = 4.15) (*t* (50) = 2.52, *P <* 0.05). When considering the two autistic subgroups divided according to their RCPM performance (5-90 RCPM, *N* = 17; below-5 RCPM, *N* = 9; see above), an ANOVA revealed that the groups significantly differed from one another on the CEFT score (*F* (2, 48) = 6.55, *P* < 0.01) with a large effect size (*η*_p_^2^ = 0.21). The Tukey HSD *post hoc* comparisons indicated that performances of nonautistic children (*M* = 18.19; SD = 4.15) and the 5-90 RCPM autistic subgroup (*M* = 16.76; SD = 3.38) did not differ significantly (*P* = 0.47), and both groups were significantly better than the below-5 RCPM autistic subgroup (*M* = 12.50; SD = 4.03) (see Figure 5).

**Figure 6:**
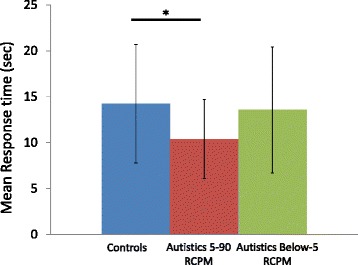
**CEFT mean response times.** Results shown are for the successful trials for the below-5 RCPM autistic subgroup (*N* = 9), the 5-90 RCPM autistic subgroup (*N* = 17), and controls (*N* = 27). Asterisk represents *P* < 0.05.

Given the equivalent scores of the 5-90 RCPM autistic subgroup and the nonautistic group, response times on successful trials could be compared (data from one autistic outlier, response time more than 3 interquartile range from the mean, was removed). Figure 6 illustrates the significant response time advantage (*t* (41) = 2.15, *P* < 0.05) of the 5-90 RCPM autistic subgroup (*M* = 10.39 seconds; SD = 4.31) over nonautistic children (*M* = 14.23 seconds; SD = 6.46).

Also, the subgroups of 13 autistics and 13 nonautistics matched on RCPM scores and age (as in the visual search section, above) were compared on the CEFT, and while both RCPM-matched autistic children (*M* = 17.62; SD = 3.38) and nonautistic children (*M* = 18.46; SD = 2.96) found the same number of figures (*P* = 0.50), RCPM-matched autistic children were significantly faster (*M* = 10.18; SD = 0.3.9) than nonautistic children (*M* = 16.43; SD = 7.66) for successful trials (*P* < 0.05).

There was a strong positive correlation between CEFT score and performance on RCPM for autistic children (*r* = 0.72, *P* < 0.001); finding more hidden figures was associated with better RCPM performance. The relation was also significant in the nonautistic group, but with a smaller correlation coefficient (*r* = 0.48, *P* < 0.05). As with visual search, correlations were done while controlling for age because there are no age-stratified norms for CEFT.

## Discussion

We piloted a school-based strength-informed assessment for autistic children with little or no spoken language and, according to school placement and conventional assessments, the highest level of impairment. That is, their cognitive potential was judged to be extremely limited. Of 30 minimally verbal school-aged autistic children, none could complete WISC-IV and only 20% (*N* = 6) could complete any WISC-IV subtest. These children would in consequence be judged as untestable, as unable to achieve a basal score, as non-evaluable due to discrepancies between subtest scores, as being to various degrees intellectually disabled, and/or as ‘low-functioning’. In contrast, 90% (*N* = 27) of these children could complete at least two of three tests in our strength-informed assessment, 83.3% (*N* = 25) could complete all three, and autistic performance was correlated across the three tests. Of the 30 reassessed autistic children, 56.7% (*N* = 17) achieved RCPM scores at or above the 5th percentile, or approximately an IQ of 75 or higher. While 13% (*N* = 4) could not be tested on RCPM, 27% (*N* = 8) were at or above the 50th percentile, and strikingly, 10% (*N* = 3) achieved an RCPM score at the 90th percentile. Correlations between autistics’ RCPM scores and their visual search or CEFT performance are evidence that perceptual tasks may be valid avenues for estimating more general cognitive potential in minimally verbal school-aged autistic children.

Our results suggest that some school-aged autistic children are at risk of being underestimated as to their cognitive potential, and that a relatively simple strength-informed assessment, compatible with low-resource settings, is a neglected approach worth pursuing. However, there is clearly room for improvement. For example, autistic children who did not perform well on or did not complete our assessment may in fact have very limited abilities, but in the alternative, their results may reflect shortcomings in our pilot effort. It was noted by the testers that minimally verbal school-aged autistic children may be trained (for example, to stack all same-shaped items, or to place a series of items into a same-shaped space, see ‘Methods’ section above) in ways which make it more difficult to accurately assess their potential. They may also experience entrenched routines and expectations, day in day out, which when disrupted (for testing by strangers, for example) understandably result in confusion and/or distress. Better ways to address these issues for those autistic children in whom they cause difficulty would improve our strength-informed assessment. In addition, we chose only three of numerous possible tasks on which autistics may excel, according to existing findings involving a very wide range of children and adults [25]. Our tasks were also narrow in scope, not assessing abilities which may be strong in some minimally verbal autistics, such as numerical abilities, receptive vocabulary, or the ability to decode text [36-38]. Nor did we take advantage of test administration using touch screens or tablets, which may be attractive to many autistic children. Further, there is some evidence that autistics may be advantaged by more complex tasks or versions of tasks, such as the adult Embedded Figures Test [39] rather than the children’s version [40], more complex versus less complex mental rotation [27], more abstract versus more concrete tests [41], and more complex versus less complex matrix reasoning problems [15]. Thus, there are many ways in which our piloted assessment may be improved upon or extended, in order to more fairly assess the potential of all autistic children.

## Conclusions

The great majority of minimally verbal autistic children could be tested, in their schools, using a strength-informed approach even as piloted here, with all stated limitations. In being tested, the autistic children in our pilot study revealed interest in the task at hand, to the point of purposefully breaking entrenched reinforcement routines; resourcefulness in using novel strategies (for example, turning the target in the CEFT); and in some, cognitive potential which may reach or exceed that of the typical population.

To conclude, we need to consider what happens to autistic children who are indeed underestimated as well as considered too old for popular interventions to meaningfully alter their outcomes [42]. Their difficult situation is made more so by prevailing views in which atypical autistic strengths and related strong interests are interpreted negatively as deficits, as suboptimal, as impediments to learning, or as symptoms which worsen autism ‘severity’ (for example, [43,44]; for more examples and reviews, see [9,45,46]). This contrasts with what is proposed about the typical population (for example, [47,48]), where any single cognitive strength, even which initially is of small magnitude, can lead to large eventual advantages via progressive access to more complex information and more demanding activities. In this model, low ability results when ‘discrimination reduces one group’s access to more cognitively demanding activities’, ([47], p.17) or when being underestimated results in a cascading loss of access to complex information and commensurate opportunities. This model may be especially relevant for autistics, due to their overall atypicality in information processing [49], with higher variance and greater specialization in preferences and abilities [50]. Thus, a strength-informed assessment in autism should be considered only a first step toward improving access to the atypical materials, information, and opportunities which allow autistic children to best develop their abilities.

## Endnote

^a^To reduce unhelpful biases (see, for example, [51]), and in keeping with the current consensus on language in autism research [52], we use the accurate, respectful terms ‘autistic’ and ‘autistic children’.

## Abbreviations

CEFT: Children’s Embedded Figure Test
RCPM: Raven’s Colored Progressive Matrices
